# Analytical Robustness and Competing Interpretations in Violent Video Game Research: A Response to Teng and Bushman's (2026) Reanalysis of Lacko et al. (2024)

**DOI:** 10.1002/ab.70073

**Published:** 2026-06-10

**Authors:** David Lacko, Hana Machackova, David Smahel

**Affiliations:** ^1^ Interdisciplinary Research Team on Internet and Society Masaryk University Brno Czech Republic

## Abstract

Recent debates on violent video game (VVG) effects highlight the importance of analytical robustness and transparency in longitudinal research. Teng and Bushman reanalyzed our prior study, identifying several methodological concerns and arguing that our conclusions were unwarranted. In this response, we examine their reanalysis and assess whether their conclusions hold across alternative analytical specifications and operational decisions. Through comprehensive robustness checks available on OSF, we address concerns regarding model specification, missing data, measurement decisions, and time constraints. We further examine the unexpected empathy–aggression association and alternative VVG operationalizations. While some inconsistencies emerge across different specifications, the results do not consistently support General Aggression Model (GAM) interpretations in this dataset. We further identify inconsistencies and incomplete reporting in Teng and Bushman's presentation of results, data processing procedures, and interpretation of effect sizes. Finally, we argue that the current debate reveals a fundamental theoretical problem: without precise specifications of mechanisms, developmental patterns, and falsifiable predictions, GAM risks unfalsifiability when contradictory findings can all be accommodated as supporting evidence. We advocate for formal modeling approaches to enhance theoretical rigor and predictive specificity in violent media research.

1

For any dataset, multiple plausible analytic approaches usually exist, and reasonable choices can lead to meaningfully different conclusions (Wagenmakers et al. 2022). Given this analytic flexibility, thorough robustness checks are essential (Aczel et al. 2026). Teng and Bushman's (“TB”; 2026) response to our article (Lacko et al. 2024) provides an opportunity to further examine the robustness and interpretation of our findings. Because TB's reanalysis reflects one set of analytical choices, we test whether their interpretation holds across alternative specifications and operational decisions. All robustness checks are available on OSF (https://osf.io/q5wv6/).

## 2 Reaction to TB's Criticism

First, we address concerns that were either unsupported or reflected matters of preference and therefore did not threaten the validity of the original findings. TB raised issues regarding our interpretation of the General Aggression Model (GAM), missing information, methodological decisions, missing data, measurement of aggression, causal inference, time constraints, and overparameterization. Their arguments, our responses, and the supporting robustness checks are summarized in Table 1.

**Table 1:** Summary of unsupported TB's concerns.

Topic	TB's argue	Our response	Details
Misunderstanding of GAM	H9–H12 were formulated as “increases in VVG are associated with decreases in aggression and increases in empathy.”	True, but this is a typographical error; the wording should be the opposite. In the rest of the article, the hypotheses are correctly interpreted.	Not needed.
Missing information	ICCs and descriptive statistics of variables are not reported.	True; we have now reported them on OSF. These show that the null within‐person effects are not artifacts of model misspecification.	OSF tab “Descriptives.”
Trait vs. state	Trait measures are not suitable; state measures should be used instead.	Disagree; given the 6‐month time window, state measures are not suitable, and trait measures are appropriate. This is common in longitudinal VVG research (cf., Teng et al. 2019, 2022).	Not needed.
Measurement of aggression	Lacko et al. initially intended to use the full BPAQ (29 items) but employed a shortened version due to translation and psychometric concerns.	Not true. We intended to use the short version (BPAQ‐SF) from the start and did not administer the full BPAQ. One additional item per subscale was included in the raw data due to concerns about how the scale would function in the Czech context, but re‐running analyses with 4 instead of 3 items per subscale did not change the patterns of the results. Thus, we used the validated set of items that is commonly used (i.e., BPAQ‐SF) rather than creating an ad hoc mix of items that would make our results more difficult to compare across existing studies.	OSF ab “BPAQ.”
Missing values	Given the high proportion of missing data, results might be biased. The data were not MCAR.	Not supported. Because MNAR cannot be formally tested and there is no evidence suggesting MNAR mechanisms, the assumption of MAR and use of FIML appear appropriate. Moreover, age, a predictor of missingness, was included as a time‐invariant covariate in the original analysis. Yet, we verified that neither using covariates as auxiliary variables in FIML nor an alternative Bayesian approach to handling missing values substantially changes the interpretation of the results.	OSF tab “Missingness.”
Time constraints	Lacko et al. did not apply any equality constraints.	Not true. While the reported model was unconstrained, we also tested (and reported on OSF) models with constraints over time on regressions, covariances, residual variances, and grand means. As stated in the article (p. 5), we chose the unconstrained model because the chi‐square difference test, recommended by Mulder and Hamaker (2021), indicated that constraining the model significantly worsened model fit. Here, we further examined whether time constraints could be applied across different operationalizations of VVG (max, sum, mean, mean × time), but in all cases, these constraints reduced model fit (*p* < 0.05). Yet, we agree with TB that, in longitudinal research, applying time constraints is good practice when empirically supported and when there is no theoretical reason to avoid them, and we recommend following this approach.	OSF tab “Time.”
Causal language	Lacko et al. used causal language repeatedly.	Partially true. Our manuscript does not repeatedly use causal language when describing results. The only instance occurs in stating the absence of evidence: “no causal evidence to support the desensitization hypothesis.” More generally, our terminology follows standard conventions in longitudinal modeling, where “causal evidence” refers to expected temporal (cross‐lagged) associations rather than experimental cause–effect inference (e.g., Orth et al. 2021; Usami 2021). While significant temporal effects provide only limited and conditional support for causal interpretations, the absence of such effects indicates that the expected temporal pattern was not observed under the present modeling framework. As we note (p. 7), “the mixed effects of physical aggression on VVG are likely not inherently stable or caused by developmental processes, as indicated by the absence of discernible trends in the data.” Importantly, absence of evidence does not constitute evidence of absence, and this statement should be interpreted in this limited sense. This distinction highlights that we are discussing the absence of evidence for causal effects under the present design, rather than making causal claims.	Not needed.
Overparameterization	Using five variables in the RI‐CLPM might lead to biased estimates due to overparameterization.	Not supported. TB selected three variables instead of five, without empirical, simulation, or theoretical support. Our model is not overparameterized; it has sufficient degrees of freedom and shows no convergence or related issues. We verified results by running two separate tripartite models for aggression and empathy. The inconsistencies were minimal. We also inspected the variance–covariance matrices of variables and model parameters, which showed no problematic correlations.	OSF tab “Parameterization.”

Next, we address arguments that we consider relevant and potentially threatening to the validity of the original results. TB argued that the positive between‐person association between affective empathy and aggression constitutes a serious issue. This concern would be warranted if the association were robust and substantial. However, meta‐analyses (cited also by TB) indicate that the relationship is weak and heterogeneous (*r* ≈ −0.10; Ritchie et al. 2022; Vachon et al. 2014), with empathy accounting for only ~1% of the variance in aggression (Vachon et al. 2014). Moreover, similar positive associations have appeared in recent studies (Kahhale et al. 2024; Palumbo and Latzman 2021). Importantly, prior studies reporting negative associations did not distinguish within‐ and between‐person variance; their findings are not directly comparable to our RI‐CLPM results. For robustness, we conducted additional analyses: testing strict measurement invariance, estimating models with only aggression and affective empathy to rule out suppressor effects, and re‐estimating the model without affective empathy (OSF tab “AMES”). None indicated problems with the empathy measure.

TB further argued that our operationalization of VVG using the maximum score across up to three games is inappropriate. Although reasonable, alternative approaches have clear drawbacks. Because we did not measure time spent per game, weighted or frequency‐based scores would confound violent content with overall gaming frequency. Mean or sum scores introduce similar inconsistencies. For example, a participant playing three games with scores 1, 2, and 2 would obtain a higher *sum* than someone playing a single game scored 4, despite lower overall violent exposure. A participant playing two highly violent games (5 and 5) and one low‐violence game (1) would have a lower *mean* than someone playing only one game scored 4. TB's suggestion that prosocial gaming offsets violence is also speculative, as low violence does not imply higher prosocial content, and vice versa. Given that GAM assumes that mere exposure to violent content can increase aggression, the maximum score remains the most defensible operationalization. Nevertheless, we re‐estimated models using alternative operationalizations (max, sum, mean, and mean × time). Some inconsistencies emerged because these indicators are correlated but not identical (*r* > 0.75). Most differences occurred in the T3 → T4 cross‐lagged paths and varied across operationalizations. Importantly, although our findings are not fully robust across VVG operationalizations, none consistently supported any GAM interpretation (desensitization or selection), nor challenged our conclusion that overall evidence for GAM is limited (OSF tab “VVG”).

TB also argue that longitudinal analyses capture participants “mid‐stream” in developmental processes, so cumulative VVG effects manifest primarily as between‐person differences, limiting the value of within‐person estimates. While theoretically interesting, this claim lacks empirical support. Longitudinal studies capable of testing cumulative within‐person effects beyond our approach are lacking, and evidence for GAM's cumulative mechanism remains limited (Drummond et al. 2020; Ferguson and Dyck 2012). Addressing this fully would require intensive burst designs and methods for modeling random slopes. Conceptually, within‐person change aligns with GAM processes, as TB acknowledge (Teng et al. 2019, 2022). GAM treats VVG as a situational input influencing proximal affective and cognitive processes, which should manifest as within‐person fluctuations.

TB's claim that cumulative processes appear mainly as between‐person variance is also problematic. Between‐person variance reflects individuals' standing during the measurement window, not earlier developmental histories. If cumulative desensitization effects accrue prior to the study, older participants should differ systematically from younger participants in VVG‐related associations. In other words, age‐related differences in desensitization would be expected. However, neither our analyses nor TB's reanalysis provided evidence of such patterns. Both analyses identified only selection effects. Moreover, in the original analysis, we found no evidence of trait‐level change (trends or growth) when examining grand means over time. We also tested mediation for VVG → empathy → aggression to capture potential cumulative effects across waves. None of the indirect effects were statistically significant (OSF tab “Mediation”).

## 3 Limitations and Inconsistencies of TB's Response

TB's reanalysis appears to rely on a subset of estimated models, leaving the full scope of results unclear. Although multiple models were estimated, only selected results were interpreted. In contrast, we interpret results across all estimated models, including inconsistencies, to provide a complete and robust interpretation. TB also evaluated time constraints using fit indices rather than the recommended chi‐square difference test (Mulder and Hamaker 2021). Even by their criteria, Model 4 (full constraints over time) should be preferred over Model 3 (partial constraints over time). Notably, in Model 4, the within‐person VVG → empathy effect (the only effect supporting GAM at the within‐person level) becomes non‐significant. This effect also disappears when T1 aggression is included as a time‐invariant covariate, suggesting it may be spurious. Re‐estimating the models using alternative VVG operationalizations confirmed this pattern: although some operationalizations produced negative within‐person effects on aggression (contrary to GAM), none yielded a significant negative effect of VVG on empathy (OSF tab “Inconsistencies”).

Further issues arise from TB's VVG computation. We identified systematic discrepancies between the means reported by TB and the correct values derived from the raw data. For example, participant 210 played games scored 2, 4, and 3 at Wave 1; the correct mean is 3, yet TB's dataset lists 4. Across participants, TB's index correlates only *r* ≈ 0.88 with the correct mean, leaving ~22% of variance unexplained (OSF tab “VVG”). Because TB did not provide the processing syntax, these discrepancies cannot be verified, and their derivation is unclear. This lack of detail in reported procedures is critical given the central role of TB's operationalization in their analyses. As shown in Table 2, relative to the original max‐score approach, TB's VVG operationalization based on unexplained data modification yields the highest proportion of non‐robust effects (i.e., deviated the most), compared to all other plausible alternatives (including in the correctly computed mean×time specification; OSF tab “Inconsistencies”). TB also interpreted effect sizes inconsistently: catharsis effects contradicting GAM were labeled small even when they exceeded the magnitude of desensitization effects considered meaningful when supporting GAM. This asymmetric application of effect size thresholds, depending on theoretical alignment, complicates interpretation and undermines the reliability of their conclusions.

**Table 2:** Proportions of non‐robust effects using different VVG operationalization under time‐constrained models.

Model	Δ*p*	Δ*β*	Δ*p* and Δ*β*
VVG as mean (correctly computed)	4 (3.8%)	3 (2.9%)	0 (0%)
VVG as sum	5 (4.8%)	4 (3.8%)	0 (0%)
VVG as mean × time	4 (3.8%)	1 (1%)	0 (0%)
VVG as mean × time used by TB^a^	9 (8.6%)	7 (6.7%)	3 (2.9%)

*Note:* All models were estimated with full constraints over time (regressions, covariances, residual variances, and grand means) and scalar measurement invariance for a clearer comparison. We applied robustness criteria similar to those used by Aczel et al. (2026). Specifically, we examined how many of (a) the interpretation of the *p*‐value (significant vs. non‐significant at the 5% threshold; Δ*p*), (b) the standardized effect size by more than ±0.05 (Δ*β*), and (c) both conditions simultaneously (Δ*p* and Δ*β*).

^a^ The last row represents comparisons with incorrect arithmetic means (see above) of VVG using data shared at OSF by TB.

## 4 Broader Implications for GAM in Future Research

This highlights a broader problem for GAM. TB repeatedly refer to meaningful effect sizes and practical significance, yet GAM provides no criteria for what is practically significant. The theory also does not specify cumulative mechanisms or how age and exposure duration shape effects. In our 2‐year study, with 6‐month intervals starting at age 11, we expect such patterns to emerge if they exist. Instead, TB argue that long‐term effects occurred before the study, implying no cumulative change should be detectable. They further suggest that negative within‐person effects of VVG on aggression align with catharsis theory. However, catharsis theory is incompatible with GAM and has been explicitly rejected by GAM proponents (Bushman et al. 1999; Bushman 2002). Such findings should therefore be interpreted as contradictory to GAM. Consequently, if null, positive, and negative effects can all be framed as evidence in support of the model, a serious epistemological problem emerges: the theory becomes unfalsifiable and thus unscientific (Devilly et al. 2023; Finkel 2014).

We acknowledge GAM's historical significance and its potential contemporary relevance, and we note that a single study cannot disprove the model, though our findings may limit its generalizability across cultural and contextual settings that may shape how violent media exposure relates to aggression. This makes cross‐context testing especially important for identifying potential boundary conditions. However, for GAM to remain scientifically useful, greater theoretical specificity is needed. Many scholars argue that addressing the broader theory crisis requires a shift toward formal modeling, which enhances rigor, transparency, and predictive specificity (Eronen and Bringmann 2021; Oberauer and Lewandowsky 2019; van Dongen et al. 2025). Such a shift would require articulating precise hypotheses, including expected effect sizes and causal pathways, thereby rendering GAM empirically testable and falsifiable. Although formal modeling demands more rigorous groundwork than traditional post hoc interpretations, it is essential for strengthening the field's predictive power and theoretical depth.

## Ethics Statement

The authors have nothing to report.

## Conflicts of Interest

The authors declare no conflicts of interest.

## Supporting information

Supporting File

## Data Availability

The data that support the findings of this study are openly available in Open Science Framework at https://osf.io/q5wv6/.

## References

[ab70073-bib-0001] Aczel, B. , B. Szaszi , H. T. Clelland , et al. 2026. “Investigating the Analytical Robustness of the Social and Behavioural Sciences.” Nature 652, no. 8108: 135–142. 10.1038/s41586-025-09844-9. 41922703

[ab70073-bib-0002] Bushman, B. J. 2002. “Does Venting Anger Feed or Extinguish the Flame? Catharsis, Rumination, Distraction, Anger and Aggressive Responding.” Personality and Social Psychology Bulletin 28, no. 6: 724–731. 10.1177/0146167202289002.

[ab70073-bib-0003] Bushman, B. J. , R. F. Baumeister , and A. D. Stack . 1999. “Catharsis, Aggression, and Persuasive Influence: Self‐Fulfilling or Self‐Defeating Prophecies?” Journal of Personality and Social Psychology 76, no. 3: 367–376. 10.1037/0022-3514.76.3.367. 10101875

[ab70073-bib-0004] Devilly, G. J. , A. Drummond , J. D. Sauer , A. Copenhaver , J. Kneer , and C. J. Ferguson . 2023. “Directional Is the New Null? A Comment on Bushman and Anderson (2021).” Psychology of Popular Media 12, no. 3: 364–372. 10.1037/ppm0000447.

[ab70073-bib-0005] Drummond, A. , J. D. Sauer , and C. J. Ferguson . 2020. “Do Longitudinal Studies Support Long‐Term Relationships Between Aggressive Game Play and Youth Aggressive Behaviour? A Meta‐Analytic Examination.” Royal Society Open Science 7, no. 7: 200373. 10.1098/rsos.200373. 32874632 PMC7428266

[ab70073-bib-0006] Eronen, M. I. , and L. F. Bringmann . 2021. “The Theory Crisis in Psychology: How to Move Forward.” Perspectives on Psychological Science 16, no. 4: 779–788. 10.1177/1745691620970586. 33513314 PMC8273366

[ab70073-bib-0007] Ferguson, C. J. , and D. Dyck . 2012. “Paradigm Change in Aggression Research: The Time Has Come to Retire the General Aggression Model.” Aggression and Violent Behavior 17, no. 3: 220–228. 10.1016/j.avb.2012.02.007.

[ab70073-bib-0008] Finkel, E. J. 2014. “The I3 Model.” In Advances in Experimental Social Psychology, 1–104. 10.1016/b978-0-12-800052-6.00001-9.

[ab70073-bib-0009] Kahhale, I. , J. L. Hanson , A. Raine , and A. L. Byrd . 2024. “Associations Between Subtypes of Empathy and Aggression in High‐Risk Adolescents.” Journal of Psychopathology and Behavioral Assessment 46, no. 1: 62–75. 10.1007/s10862-023-10112-1. 40837349 PMC12364107

[ab70073-bib-0010] Lacko, D. , H. Machackova , and D. Smahel . 2024. “Does Violence in Video Games Impact Aggression and Empathy? A Longitudinal Study of Czech Adolescents to Differentiate Within‐ and Between‐Person Effects.” Computers in Human Behavior 159, no. 108341: 108341. 10.1016/j.chb.2024.108341.

[ab70073-bib-0011] Mulder, J. D. , and E. L. Hamaker . 2021. “Three Extensions of the Random Intercept Cross‐Lagged Panel Model.” Structural Equation Modeling: A Multidisciplinary Journal 28, no. 4: 638–648. 10.1080/10705511.2020.1784738.

[ab70073-bib-0012] Oberauer, K. , and S. Lewandowsky . 2019. “Addressing the Theory Crisis in Psychology.” Psychonomic Bulletin & Review 26, no. 5: 1596–1618. 10.3758/s13423-019-01645-2. 31515732

[ab70073-bib-0013] Orth, U. , D. A. Clark , M. B. Donnellan , and R. W. Robins . 2021. “Testing Prospective Effects in Longitudinal Research: Comparing Seven Competing Cross‐Lagged Models.” Journal of Personality and Social Psychology 120, no. 4: 1013–1034. 10.1037/pspp0000358. 32730068 PMC7854859

[ab70073-bib-0014] Palumbo, I. M. , and R. D. Latzman . 2021. “Parsing Associations Between Dimensions of Empathy and Reactive and Proactive Aggression.” Journal of Personality Disorders 35, no. Supple C: 56–74. 10.1521/pedi_2021_35_522. 33999656

[ab70073-bib-0015] Ritchie, M. B. , R. W. J. Neufeld , M. Yoon , A. Li , and D. G. V. Mitchell . 2022. “Predicting Youth Aggression With Empathy and Callous Unemotional Traits: A Meta‐Analytic Review.” Clinical Psychology Review 98: 102186. 10.1016/j.cpr.2022.102186. 36240695

[ab70073-bib-0016] Teng, Z. , and B. J. Bushman . 2026. “Best Practice Procedures in Longitudinal Research of Violent Media Effects: Reanalysis of the Lacko et al. (2024) Study as a Case Study.” Aggressive Behavior 52, no. 1: e70054. 10.1002/ab.70054. 41467508

[ab70073-bib-0017] Teng, Z. , Q. Nie , C. Guo , Q. Zhang , Y. Liu , and B. J. Bushman . 2019. “A Longitudinal Study of Link Between Exposure to Violent Video Games and Aggression in Chinese Adolescents: The Mediating Role of Moral Disengagement.” Developmental Psychology 55, no. 1: 184–195. 10.1037/dev0000624. 30335433

[ab70073-bib-0018] Teng, Z. , C. Yang , M. Stomski , Q. Nie , and C. Guo . 2022. “Violent Video Game Exposure and Bullying in Early Adolescence: A Longitudinal Study Examining Moderation of Trait Aggressiveness and Moral Identity.” Psychology of Violence 12, no. 3: 149–159. 10.1037/vio0000424.

[ab70073-bib-0019] Usami, S. 2021. “On the Differences Between General Cross‐Lagged Panel Model and Random‐Intercept Cross‐Lagged Panel Model: Interpretation of Cross‐Lagged Parameters and Model Choice.” Structural Equation Modeling: A Multidisciplinary Journal 28, no. 3: 331–344. 10.1080/10705511.2020.1821690.

[ab70073-bib-0020] Vachon, D. D. , D. R. Lynam , and J. A. Johnson . 2014. “The (Non)Relation Between Empathy and Aggression: Surprising Results From a Meta‐Analysis.” Psychological Bulletin 140, no. 3: 751–773. 10.1037/a0035236. 24364745

[ab70073-bib-0021] van Dongen, N. , R. van Bork , A. Finnemann , et al. 2025. “Productive Explanation: A Framework for Evaluating Explanations in Psychological Science.” Psychological Review 132, no. 2: 311–329. 10.1037/rev0000479. 39023936

[ab70073-bib-0022] Wagenmakers, E. J. , A. Sarafoglou , and B. Aczel . 2022. “One Statistical Analysis Must Not Rule Them All.” Nature 605, no. 7910: 423–425. 10.1038/d41586-022-01332-8. 35581494

