# Supplementary material for: Analytical Robustness and Competing Interpretations in Violent Video Game Research: A Response to Teng and Bushman's (2026) Reanalysis of Lacko et al. (2024)
Source: Aggress Behav. 2026 Jun 10;52(4):e70073. doi: 10.1002/ab.70073 (PMC13250609; doi:10.1002/ab.70073)
Supplement: Supplementary file 1 — Supporting File [file AB-52-e70073-s001.docx]

# **Supplementary Materials**

The following supplementary materials represent summarized versions of the analyses presented in the OSF repository. Each subsection (referred to in the main text as OSF tabs) provides a brief overview of the corresponding analyses to improve accessibility for a broader readership. Full analytic details, code, and all supporting results remain available on OSF (<https://osf.io/q5wv6/overview>).

## **Descriptives**

We calculated intraclass correlation coefficients (ICCs) for all main variables to quantify the proportion of variance attributable to stable between-person differences versus within-person fluctuations across the four waves. The ICCs were moderate for all constructs:

- cognitive empathy (ICC = 0.53),
- affective empathy (ICC = 0.47),
- violent content exposure (ICC = 0.55),
- verbal aggression (ICC = 0.49),
- and physical aggression (ICC = 0.58).

These values indicate that approximately half of the total variance lies between individuals, with the remaining variance reflecting within-person changes over time. Importantly, these ICCs confirm that there is sufficient within-person variability to justify the use of a random-intercept cross-lagged panel model (RI-CLPM). Neither extremely high (> .80) nor extremely low (< .10) ICCs were observed, suggesting that the null within-person effects reported are unlikely to be artifacts of an inappropriate model specification.

## **Missingness**

Although we had no indication that our data followed non-ignorable (MNAR) missingness patterns beyond the typical longitudinal dropout structure, and no substantively meaningful variables were available to explain missingness mechanisms, we conducted several robustness checks. In the attrition analysis, age emerged as a significant predictor of dropout. We therefore included age as a time-invariant covariate in the RI-CLPM—both to account for its effects and to allow interpretation of age-related differences in the random intercepts in the original analysis.

To evaluate the robustness of the findings to missing data handling, we re-estimated the RI-CLPM using a Bayesian framework with eight MCMC chains (Gibbs sampling, 25,000 iterations per chain, thinning = 10). For computational stability in this large multivariate model, indicator error variances were constrained to small positive values (rather than fixed to zero), a standard practice that improves MCMC efficiency without altering substantive interpretations. The Bayesian model exhibited no convergence issues: all potential scale reduction factors (PSR) were below 1.10, and visual inspection of trace plots, posterior distributions, and autocorrelation diagnostics revealed no trends or anomalies. The complete Mplus output is available on OSF (see *ri-clpm_bayes.out*).

Bayesian estimation, which handles missingness through posterior-based integration over missing values, yielded results that were nearly identical to those reported using FIML in the article. Because Bayesian models do not use p-values, we interpreted effects as “significant” when their 95% credible intervals excluded zero. Only two discrepancies emerged:

- (1) the effect of affective empathy T3 → cognitive empathy T4 became credible in the Bayesian model (but was non-significant under FIML; Δβ = .053),
- and (2) the effect of verbal aggression T3 → T4 became non-credible in Bayesian estimation (Δβ < .05).

Neither of these paths was part of our preregistered hypotheses, and both were small in magnitude, so they do not affect the interpretation of our main findings.

We also re-estimated all models in a frequentist framework using FIML with gender and age treated as auxiliary variables rather than covariates. This yielded two differences in cross-lagged effects and three differences at the between-person level (based on either significance or effect-size differences > .05). Specifically:

- physical aggression T1 → VVG T2
- and verbal aggression T3 → T4 changed significance status,

and several random intercept correlations shifted signs or significance. Specifically:

- RI cognitive empathy ↔︎ RI physical aggression: the correlation became negative and significant (it was nonsignificant in the main model).
- RI affective empathy ↔︎ RI violent gaming: the correlation became negative and significant (it was nonsignificant in the main model).
- RI affective empathy ↔︎ RI physical aggression: the correlation became nonsignificant (it was significant in the main model).

However, this auxiliary-variable model produced a non–positive definite residual covariance matrix (a Heywood-type problem), indicating that these estimates are likely biased and should not be interpreted.

Taken together, Bayesian estimation (which produced a well-behaved model) replicated the original findings almost fully, and the FIML auxiliary-variable model showed only minor discrepancies among 105 total estimated paths. Thus, we conclude that our results are robust with respect to missing-data handling, with one exception: the verbal aggression T3 → T4 effect, which was borderline in the original model (p = .048) and consistently non-significant across both alternative missingness approaches. This path was not part of our hypotheses and does not affect any substantive conclusions.

## **BPAQ**

Regarding the robustness of the Buss–Perry aggression scales, we re-estimated the RI-CLPM using four items per subscale instead of the original three-item shortened version. This comparison revealed seven inconsistencies in cross-lagged effects according to significance criteria, with only one of them showing an effect-size difference greater than .05. Specifically:

- WF_cog_empathy_W1 → WF_verbal_agg_W2 became significant (p = .043).
- WF_physic_agg_W2 → WF_cog_empathy_W3 became nonsignificant (p = .074).
- WF_verbal_agg_W2 → WF_verbal_agg_W3 became significant (p = .011).
- WF_aff_empathy_W2 → WF_physic_agg_W3 became nonsignificant (originally p = .045).
- WF_VC_W4 → WF_physic_agg_W3 became nonsignificant (p = .052).
- WF_verbal_agg_W4 → WF_physic_agg_W3 became nonsignificant (p = .060).
- WF_physic_agg_W4 → WF_physic_agg_W3 became nonsignificant and showed the only notable decrease in effect size (> .05).

All of these changes were small in absolute magnitude, and in terms of statistical significance, both the original and alternative estimates were consistently near the conventional α = .05 threshold. Importantly, all inconsistencies except one involved non-hypothesized paths. The single exception (WF_VC_W4 → WF_physic_agg_W3) exhibited a negligible effect-size change (ΔES = .011), which we still consider robust. Finally, it is important to underscore that the four-item version of each Buss–Perry subscale is not validated, neither in the original literature nor in the Czech context. Therefore, these alternative estimates should be viewed as less reliable, and our primary results based on the standard shortened version remain the most appropriate and valid for interpretation.

## **AMES**

Due to the unexpected positive correlation between affective empathy and aggression at the between-person level, TB argued that the AMES measure might be invalid. To address this, we carefully compared our estimates of reliability, factor loadings, and subscale correlations to the original AMES scale and found no notable discrepancies. For completeness, we also tested strict measurement invariance over time, which was successfully established. This level of invariance ensures that factor loadings, item intercepts, and residual variances are equal across waves, indicating that the construct is measured consistently over time and that observed changes reflect true within-person variability rather than measurement artifacts.

Next, we investigated whether the unexpected effect could be caused by a potential suppressor effect by estimating a simplified model including only affective empathy and verbal and physical aggression. The correlation remained stable, with no inconsistencies at the between-person level, suggesting it is not a spurious correlation caused by suppression.

As an additional robustness check, we removed affective empathy entirely from the RI-CLPM to examine whether other effects might be confounded by potentially inappropriate measurement. This produced three minor inconsistencies in cross-lagged effects, all near the α = .05 threshold and with negligible effect-size differences (ΔES < .05):

- WF_physic_agg_W1 → WF_physic_agg_W2 became nonsignificant (p = .069)
- WF_physic_agg_W3 → WF_VC_W4 became nonsignificant (p = .062)
- WF_verbal_agg_W3 → WF_verbal_agg_W4 became nonsignificant (p = .052).

Interestingly, the last path (WF_verbal_agg_W3 → WF_verbal_agg_W4) consistently failed to replicate, suggesting that this specific effect is indeed not robust. The only path related to a hypothesized effect was the second one, but its effect-size difference was minimal (ΔES = .008). Overall, these robustness checks indicate that affective empathy is measured reliably in our data, and there is no reason to consider it invalid.

## **Parametrization**

Since TB argued that our original model might be overparameterized, we conducted robustness checks by comparing the original results with two separate trivariate RI-CLPMs, one for empathy and one for aggression. Across 210 relevant paths, we identified only four inconsistencies (three cross-lagged effects and one between-person association), all based on significance. Importantly, both the original and new p-values were near the 5% threshold in all cases, and effect-size differences were minimal (ΔES < .05). Specifically:

- In the empathy model, RI cognitive empathy ↔︎ RI violent content became nonsignificant (p = .062).
- In the aggression model, three cross-lagged paths became nonsignificant: WF_physic_agg_W1 → WF_physic_agg_W2 (p = .068), WF_physic_agg_W3 → WF_VC_W4 (p = .058), and WF_verbal_agg_W3 → WF_verbal_agg_W4 (p = .052).

Only two of these paths corresponded to hypothesized effects, and the changes in effect size were negligible. The one exception was WF_verbal_agg_W3 → WF_verbal_agg_W4, which showed somewhat lower robustness in the missingness analysis (see Table “Missingness”).

Inspection of the variance–covariance matrices at both the observed and latent levels, as well as the variance–covariance matrix of model parameters, did not reveal any large correlations, suggesting a lack of multicollinearity. Similarly, standard errors remained largely unchanged across all models. Additionally, we tested time-constrained models similarly to those used by TB. These models produced no inconsistencies, confirming that our findings are not biased due to overparameterization.

## **VVG**

Given that TB raised plausible points regarding the operationalization of VVG, we re-ran the main model using alternative operationalizations (max, sum, mean, mean × time). These models naturally yielded different estimates, as the indicators, although highly correlated (r > .75), are not identical and carry slightly different information. Since these inconsistencies were larger than in other robustness checks, we focus here only on hypothesized paths. Out of 420 relevant paths, 144 were hypothesized, and 15 inconsistencies were observed, with only 4 showing effect-size differences > .05. Most inconsistencies involved cross-lagged paths from T3 to T4.

More specifically:

- Mean score: WF_VC_W2 → WF_physic_agg_W3 became significant (p = .024), WF_aff_empathy_W3 → WF_VC_W4 became nonsignificant with a larger decrease in ES, but the magnitude of the original effect was unchanged, WF_physic_agg_W3 → WF_VC_W4 became nonsignificant (p = .084), WF_VC_W3 → WF_cog_empathy_W4 became significant (p = .016), and between-person association RI_cog_empathy ↔︎ RI_VC became significant.
- Sum score: WF_physic_agg_W1 → WF_VC_W2 became nonsignificant (p = .169), WF_cog_empathy_W2 → WF_VC_W3 became significant (p = .020), WF_aff_empathy_W3 → WF_VC_W4 became nonsignificant with a large ES change, but effect magnitude unchanged, WF_verbal_agg_W3 → WF_VC_W4 became significant with a large ES change, but effect magnitude unchanged.
- Mean × time: WF_physic_agg_W1 → WF_VC_W2 became nonsignificant with a large ES change, magnitude unchanged, WF_cog_empathy_W2 → WF_VC_W3 became significant (p = .020), WF_aff_empathy_W3 → WF_VC_W4 became nonsignificant (p = .089), WF_VC_W3 → WF_cog_empathy_W4 became significant (p = .013), WF_VC_W3 → WF_verbal_agg_W4 became significant (p = .024), and between-person association RI_cog_empathy ↔︎ RI_VC became nonsignificant.

Importantly, out of the 144 hypothesized paths, only 15 showed any inconsistencies based on p-values, and only 4 based on effect-size differences. Despite these minor inconsistencies, we did not observe a temporally stable desensitization effect. In fact, the mean and mean × time scores suggested results more consistent with catharsis theory for T3 → T4 aggression and with GAM for cognitive empathy, while the sum score provided no evidence for desensitization. Given that these unstable results were restricted to a single wave, and considering that the max score was pre-selected as the most appropriate operationalization, we conclude that these alternative operationalizations do not meaningfully alter the interpretation of our main findings. Yet, we also acknowledge that our results are not fully robust towards different VVG operationalizations.

## **Time**

Temporal constraints were tested using chi-square difference tests, comparing the original unconstrained model with models in which regressions, covariances (innovations), residual variances, and grand means were constrained to be equal over time. These tests were applied not only to the original full model, but also to two separate trivariate models for aggression and empathy, as well as to all alternative operationalizations of violent video-game exposure (VVG) - including max, sum, mean, mean × time, and the TB score. Across all comparisons, constraining the models in time significantly worsened model fit, indicating that temporal invariance assumptions do not hold for our data. Notably, this was true even for the score used by TB (see tab “Inconsistencies”). These results suggest that our original unconstrained model is appropriate and valid, and that the approach advocated by TB is not suitable for these data.

## **Mediation**

We conducted a mediation analysis to examine whether violent video game (VVG) exposure influenced verbal and physical aggression through cognitive and affective empathy across consecutive waves. Individual indirect effects were calculated for each pathway (cognitive/affective empathy → verbal/physical aggression) over two intervals: W1→W2→W3 and W2→W3→W4. Total indirect effects were computed as the sum of cognitive and affective empathy pathways, and overall total effects included both the direct effect of VVG and all mediated pathways. To ensure robust estimates, we employed 10,000 bootstrap iterations.

Across all models, no individual indirect effect, total indirect effect, or overall total effect reached significance. This indicates that neither cognitive nor affective empathy mediated the effects of VVG exposure on verbal or physical aggression during the study period.

These findings reinforce the main analyses, showing that the lack of within-person effects of VVG on aggression is not attributable to untested mediation through empathy. Even when considering multiple sequential pathways and aggregating effects across waves, no evidence of longitudinal influence via empathy emerged, supporting the robustness of our conclusions.

## **Inconsistencies**

Several inconsistencies in TB’s analysis were also identified. First, when we re-ran our models using alternative VVG operationalizations (mean, sum, mean × time) and applied time constraints to align more closely with TB’s approach, we observed only effects against GAM—i.e., increases in VVG led to decreases in aggression. None of these models provided any evidence supporting GAM as reported by TB (i.e., increases in VVG leading to decreases in empathy).

After inspecting TB’s data, we found inconsistencies in their VVG scores, which did not match the scores in our dataset (for correlations, see tab “VVG”). Surprisingly, under full-time constraints and relative to the max-score–based model, TB’s VVG score deviated the most from all other plausible operationalizations.

To further examine their model, we re-ran TB’s original Mplus model using their data, adding only a time-invariant predictor of aggression from T1; the effect then diminished (see *aggression_control.out*). Similarly, when TB themselves constrained residual covariances to be equal across time (Model 4), the effect also diminished. Thus, we are convinced that at the within-person level, if time constraints are applied (that are not justified by a chi-square difference test) and a less appropriate VVG indicator is used, one can produce evidence against GAM (in line with catharsis theory). However, the effect TB reported in favor of GAM is not robust and appears to result from selective reporting. As seen in model comparisons, their index deviates most from the original scores, even though it should have been identical to the mean × time calculation.
